# Impact of Ultraviolet C Radiation on Male Fertility in Rats: Suppression of Autophagy, Stimulation of Gonadotropin-Inhibiting Hormone, and Alteration of miRNAs

**DOI:** 10.3390/ijms26010316

**Published:** 2025-01-01

**Authors:** Ahmed Mohamed Alahwany, Ahmed Hamed Arisha, Adel Abdelkhalek, Tarek Khamis, Taku Miyasho, Doaa Kirat

**Affiliations:** 1 Department of Animal Physiology and Biochemistry, Faculty of Veterinary Medicine, Badr University in Cairo (BUC), Badr City 11829, Egypt; ahmed.mohamed8@buc.edu.eg (A.M.A.); vetahmedhamed@zu.edu.eg (A.H.A.); 2 Department of Physiology, Faculty of Veterinary Medicine, Zagazig University, Zagazig 44519, Egypt; 3 Faculty of Veterinary Medicine, Badr University in Cairo (BUC), Badr City 11829, Egypt; adel.abdelkhalek@buc.edu.eg; 4 Department of Pharmacology, Faculty of Veterinary Medicine, Zagazig University, Zagazig 44519, Egypt; t.khamis@vet.zu.edu.eg; 5 Laboratory of Animal Biological Responses, Department of Veterinary Medicine, Rakuno Gakuen University, Ebetsu 069-8501, Japan

**Keywords:** male fertility, oxidative stress, miR-20a-5p, miR-137-3p, hesperidin, molecular docking

## Abstract

While ultraviolet C (UVC) radiation has beneficial applications, it can also pose risks to living organisms. Nevertheless, a detailed assessment of UVC radiation’s effects on mammalian male reproductive physiology, including the underlying mechanisms and potential protective strategies, has not yet been accomplished. This study aimed to examine the critical roles of oxidative stress, autophagy, reproductive hormonal axis, and microRNAs in UVC-induced reproductive challenges in male rats. Semen, biochemical, molecular, and in silico analyses revealed significant dysregulation of testicular steroidogenesis, impaired spermatogenesis, deteriorated sperm quality, and altered reproductive hormonal profiles, which ultimately lead to a decline in fertility in male rats exposed to UVC radiation. Our data indicated that the suppression of autophagy, stimulation of gonadotropin-inhibiting hormone (GnIH), and alteration of microRNAs serve as key mediators of UVC-induced stress effects in mammalian reproduction, potentially contributing to male infertility. Targeting these pathways, particularly through pretreatment with hesperidin (HES), offers a promising strategy to counteract UVC-induced male infertility. In conclusion, the present findings emphasize the importance of understanding the molecular mechanisms behind UVC-induced male infertility and offer valuable insights into the protective mechanisms and prospective role of HES in safeguarding male reproductive health.

## 1. Introduction

A healthy male reproductive system demands appropriate testosterone secretion and sperm production. Several factors can affect male reproductive health, including genetic and epigenetic abnormalities, environmental pollution, use of pesticides, harmful chemicals, endocrine disruptors, lifestyle aspects, infections, exposure to excessive heat, and radiation [1,2]. When any of these factors disrupt the normal functioning of the male reproductive system, they can lead to infertility [2,3,4]. In recent years, the World Health Organization (WHO) has reported a global increase in the prevalence of male infertility, with millions of men being affected worldwide [5]. Therefore, it is necessary to investigate the factors impacting male fertility to enhance management strategies.

Numerous findings describe the detrimental consequence of radiation on the physiological parameters of the male reproductive system [2,6]. Studies advocate the thesis that the direct or diffused exposure of testicles to ionizing or non-ionizing radiations emitted from sources like cell phones, microwave ovens, laptops, X-rays, and γ-rays exerts damaging effects on the male reproductive system [2].

Ultraviolet (UV) radiation is categorized into three spectra based on wavelength: UVC (100–280 nm), UVB (280–315 nm), and UVA (315–400 nm) [7]. Ultraviolet radiation has beneficial and harmful effects depending upon the type of wavelength region and irradiation dose (intensity or duration) [7]. UVC radiation has more detrimental effects on living beings than those associated with UVA or UVB [8]. Fortunately, most UVC radiation is absorbed by the Earth’s ozone layer [9]. However, the recent thinning of this layer raises concerns that increased exposure to ultraviolet radiation could lead to numerous harmful effects on human and animal physiology, potentially resulting in reproductive defects linked to ozone depletion and global warming [10].

Artificial sources of UVC radiation, such as germicidal lamps, are used in sterilization and disinfection processes because they destroy the DNA and RNA of microorganisms. Lately, Ref. [11] deduced many environmental and health safety issues concerning the use of UVC irradiation in disinfection, and they reported the potential risks of damaging skin and eyes and even possible carcinogenic effects. Moreover, they suggested using a UVC disinfection system that does not require human intervention or wearing UVC light protection equipment to prevent these risks [11].

Concerning the effect of UV radiation on male reproduction, studies have shown an association between both UVA and UVB radiation and male infertility. However, there have been only two studies to date that examined the effect of UVC exposure on human sperm in vitro; these showed limited parameters such as an increase in lipid peroxidation and decreases in sperm motility and viability [12] as well as disorganization in the sperm chromatin and fragmentation of DNA [13]. Despite these findings, a comprehensive assessment of UVC radiation’s impact on mammalian male reproductive physiology remains to be conducted.

Recently, there has been growing evidence that autophagy is a prerequisite for male fertility because it affects several aspects of the male reproductive system. Autophagy is a lysosome-mediated intracellular degradation pathway in which unwanted cargo such as old or damaged organelles, as well as unneeded proteins, are sequestrated into autophagosomes and then delivered to the lysosomes for degradation [14]. Research studies have revealed that autophagy has roles in spermatogenesis, sperm motility, acrosome biogenesis, testosterone biosynthesis, and testicular cell integrity [14]. However, the role of autophagy in the consequential impacts of ultraviolet radiation on the male reproductive physiology is largely unexplored.

MicroRNAs (miRNAs) are short non-coding RNAs (22–24 nucleotides in length) that act as crucial regulators for post-transcriptional gene silencing by base-pairing with the 3′-untranslated regions (UTRs) of target messenger RNAs to form RNA duplexes, which leads to either the inhibition of the target mRNA or the suppression of the translation [15]. Epigenetic regulation modulated by miRNAs of different genes’ expression is a recently described mechanism widely involved in diverse physiological processes and disease conditions. Accumulating evidence has revealed that many miRNAs play key roles in male infertility [16]. Nevertheless, the potential relevance of epigenetic regulation and emerging roles of miRNAs in male fertility, autophagy process, oxidative stress, and radiation response have not yet been described under the effect of UVC radiation.

Natural plant products and phytochemicals have been used as radioprotective agents. Hesperidin (HES) is a major flavanone glycoside found in citrus fruits (lemons and sweet oranges), tea, and olive oil, and it is used in traditional Chinese medicine to treat a wide array of diseases [17]. It has many biological effects, including anti-cancer, anti-inflammatory, and antioxidant effects [18]. While HES possesses radioprotective properties in various measurement systems [17,19], nothing is known about its role in UVC radiation exposure.

The present study aimed to comprehensively investigate the effects of (UVC) radiation on male reproductive physiology and describe the molecular regulatory mechanisms that underlie the impacts of UVC radiation by emphasizing the roles of oxidative stress, autophagy, reproductive hormones, and microRNAs in UVC-induced reproductive challenges in male rats. Furthermore, this study explored the effects of HES pretreatment on UVC-irradiated male rats. Furthermore, the present study explored the effects of HES pretreatment on UVC-irradiated male rats.

## 2. Results

### 2.1. Effects of UVC Irradiation, Alone or in Combination with HES, on Testicular Weight and Gonadosomatic Index in Male Rats

**Figure 1:**
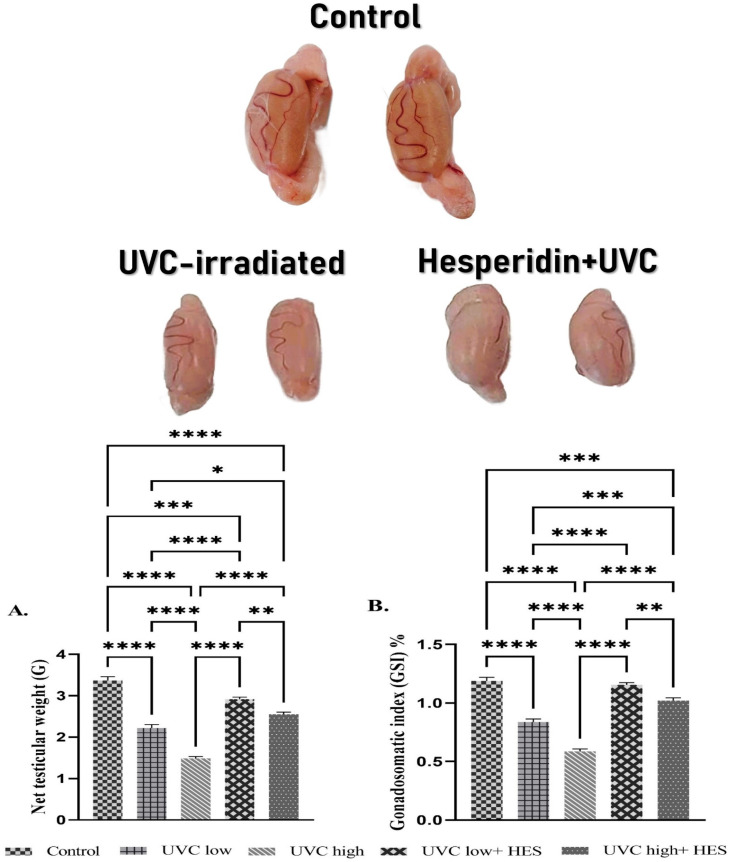
Effects of UVC irradiation, alone or in combination with hesperidin, on testicular weight (**A**) and gonadosomatic index (**B**) in male rats. Rats exposed to total body irradiation with low or high doses of artificial UVC for 8 h/day for 8 consecutive weeks showed a dose-dependent reduction in testicular weight and Gonadosomatic Index. HES alleviates the decrease of testicular weight and gonadosomatic index caused by UVC radiation. A gross morphology photograph of rat testicles is shown above the bar charts. Data are expressed as mean ± SE from six rats per group. Asterisks indicate a statistically significant difference: **** *p* < 0.0001, *** *p* < 0.001, ** *p* < 0.01, * *p* < 0.05.

At the end of the experimental period, the weight of both testicles was significantly reduced in the irradiated rats with or without HES treatment compared to the control group (Figure 1A). However, testicular weight in HES-pretreated irradiated groups was significantly increased compared to the UVC-irradiated groups (Figure 1A). Among the UVC-irradiated groups, rats that received high-dose treatments showed a significant decline in testicular weight compared to those of the low-dose-irradiated group (Figure 1A). Also, within the HES-pretreated irradiated groups, low-dose-UVC-irradiated rats pretreated with HES exhibited significant increases in the gonadosomatic index compared to the HES-pretreated high-dose irradiation group (Figure 1A).

As shown in Figure 1B, the gonadosomatic index significantly decreased in the UVC-irradiated groups compared to the control group. Amongst the UVC-irradiated groups, rats that received high doses showed a significant decline in the gonadosomatic index compared to those of the low-dose-irradiated group (Figure 1B). Also, within the HES-pretreated irradiated groups, low-dose-UVC-irradiated rats pretreated with HES exhibited significant increases in the gonadosomatic index compared to the HES-pretreated high-dose irradiation group (Figure 1B). However, the gonadosomatic index was significantly increased in HES-pretreated irradiated groups compared to the UVC-irradiated groups (Figure 1B).

### 2.2. Effects of UVC Irradiation, Alone or in Combination with HES, on the Sperm Quality of Rats

**Figure 2:**
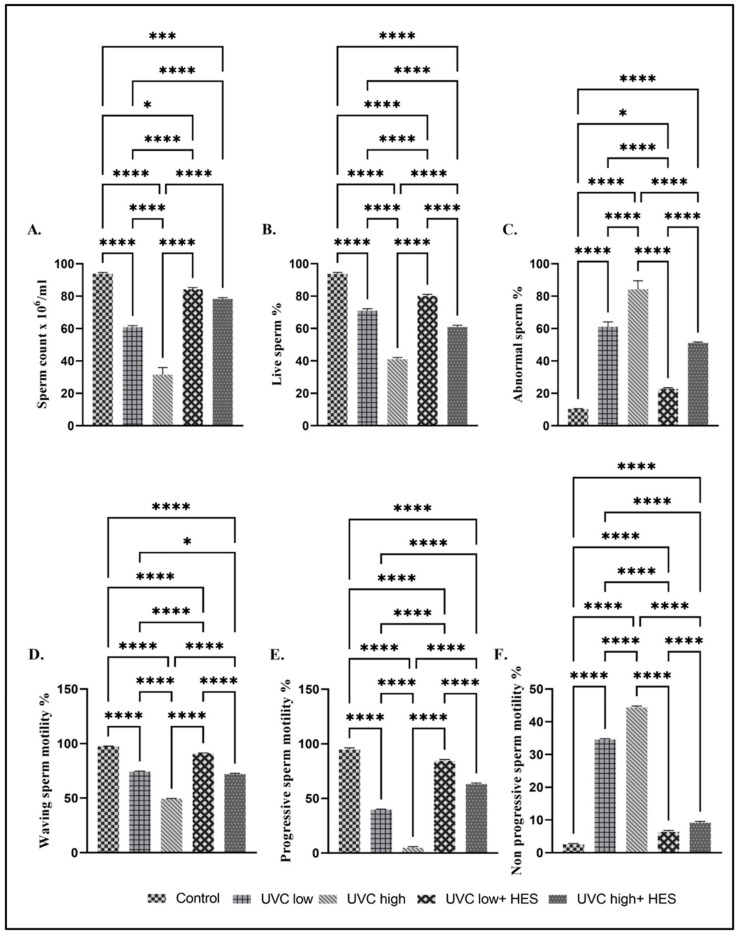
Effects of UVC irradiation, alone or in combination with HES, on the rat spermiogram. Sperm quality parameters: (**A**) sperm count, (**B**) live sperm%, (**C**) abnormal sperm%, and (**D**–**F**) sperm motility%. Data are expressed as mean ± SE from six rats per group. Statistically significant at **** *p* < 0.0001, *** *p* < 0.001, * *p* < 0.05.

The UVC-irradiation alone significantly reduced the sperm count and live sperm percentage, as well as significantly elevated the abnormal sperm percentage compared to that in the control group (Figure 2A–C). These results were also noticed in the high-dose-UVC-irradiated group compared to the low-dose-UVC-irradiated group (Figure 2A–C). On the contrary, HES-pretreated irradiated groups exhibited significant increases in the sperm count and live sperm percentage and declines in the abnormal sperm percentage compared to the control and UVC-irradiated groups (Figure 2A–C).

The UVC-irradiated groups and the HES-pretreated irradiated rats revealed significant declines in waving and progressive sperm motility and marked elevations in the non-progressive sperm motility relative to those in the control group (Figure 2D–F). Similarly, among the UVC-irradiated groups, rats that received high-dose treatments showed a significant decline in waving and progressive sperm motility, along with a noticeable increase in the non-progressive sperm motility, compared to those of the low-dose-irradiated group (Figure 2D–F). In contrast, HES-pretreated irradiated groups showed significant increases in both waving and progressive sperm motility, and marked decreases in non-progressive sperm motility, compared to the UVC-irradiated groups (Figure 2D–F).

**Figure 3:**
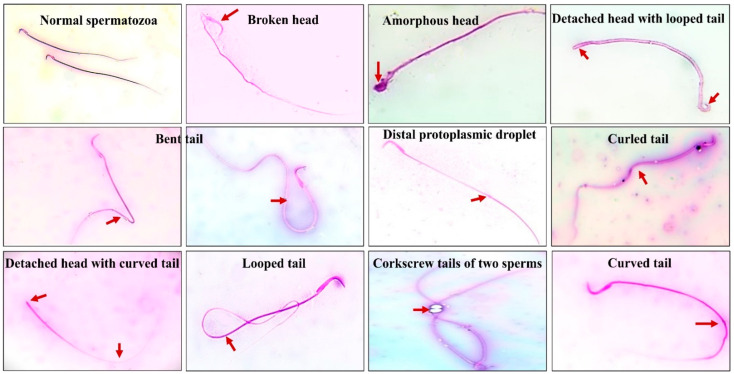
Sperm-related morphological abnormalities of rats exposed to UVC radiation. Photographs of different types of sperm abnormalities were assessed in eosin–nigrosin stained smears and viewed under the oil-immersion objective of a light microscope. Spermatozoa in all photographs are shown at ×100 magnification.

As shown in Figure 3, several sperm morphological abnormalities were detected in rats exposed to UVC radiation, including a looped tail, detached head, detached tail, curved tail, coiled tail, protoplasmic droplet, broken head, bent tail, and amorphous head.

### 2.3. Effects of UVC Irradiation, Alone or in Combination with HES, on Oxidative Stress Biomarkers in Male Rat Testes

**Figure 4:**
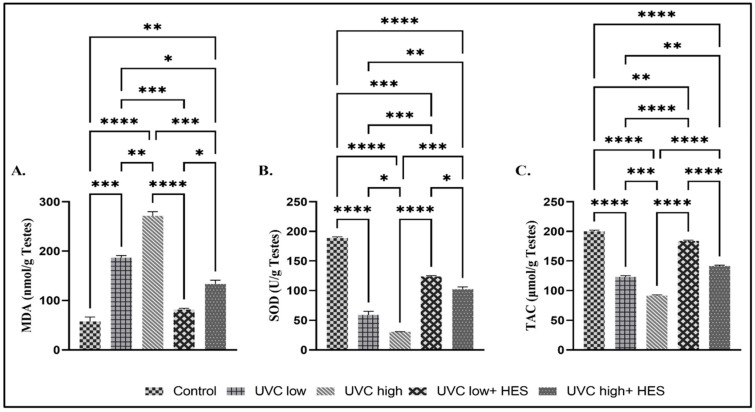
Effects of UVC irradiation, alone or in combination with HES, on oxidative stress biomarkers in male rat testes. Oxidative stress biomarkers, namely, (**A**) malondialdehyde (MDA), (**B**) superoxide dismutase (SOD), and (**C**) total antioxidant capacity (TAC), were evaluated and are represented as mean ± SE. For each group, (*n* = 6). Asterisks indicate a statistically significant difference: **** *p* < 0.0001, *** *p* < 0.001, ** *p* < 0.01, * *p* < 0.05.

Rats irradiated with UVC exhibited a significant increase in the testicular concentration of MDA relative to the control group (Figure 4A). Additionally, a marked increase in the MDA content was detected in the testicles of the high-dose-UVC-irradiated group compared to the low-dose-UVC-irradiated group, in a dose-dependent manner (Figure 4A). In contrast, HES-pretreated irradiated groups showed a significant decline in MDA levels compared to the UVC-irradiated groups (Figure 4A). Additionally, MDA levels in low-dose-UVC-irradiated rats pretreated with HES were determined to be near the level observed in the control (Figure 4A).

The testicular concentrations of SOD and TAC showed significant dose-dependent decreases in the UVC-irradiated rats, in comparison to the control group (Figure 4B,C). A significant reduction was also noticed in both SOD and TAC levels in the high-dose-UVC-irradiated group, compared to the low-dose-UVC-irradiated group (Figure 4B,C). On the other hand, levels of SOD and TAC displayed significant elevation in the testicles of HES-pretreated-UVC-irradiated groups compared to the UVC-irradiated groups (Figure 4B,C).

### 2.4. Effects of UVC Irradiation, Alone or in Combination with HES, on Serum Levels of Reproductive Hormones in Male Rats

**Figure 5:**
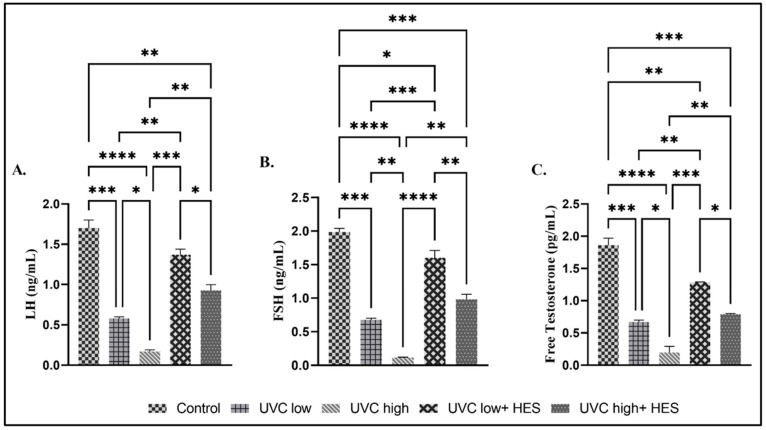
Effects of UVC irradiation, alone or in combination with HES, on serum levels of reproductive hormones in male rats. The levels of (**A**) luteinizing hormone (LH), (**B**) follicle stimulating hormone (FSH), and (**C**) testosterone were determined in the sera of control and UVC-irradiated rats with or without HES treatment. Values represent the mean ± SE from six rats per group. Asterisks indicate a statistically significant difference: **** *p* < 0.0001, *** *p* < 0.001, ** *p* < 0.01, * *p* < 0.05.

The serum concentrations of LH, FSH, and free testosterone showed significant dose-dependent decreases in the UVC-irradiated rats, relative to the control group (Figure 5A–C). A significant reduction was also noticed in LH, FSH, and free testosterone serum levels in the high-dose-UVC-irradiated group compared to the low-dose-UVC-irradiated group (Figure 5A–C). Conversely, HES-pretreated-UVC-irradiated groups displayed significant increase in the serum levels of LH, FSH, and free testosterone relative to the UVC-irradiated groups (Figure 5A–C).

### 2.5. Effects of UVC Irradiation, Alone or in Combination with HES, on the Expression Levels of Reproductive-Related Genes in the Hypothalamus of Male Rats

**Figure 6:**
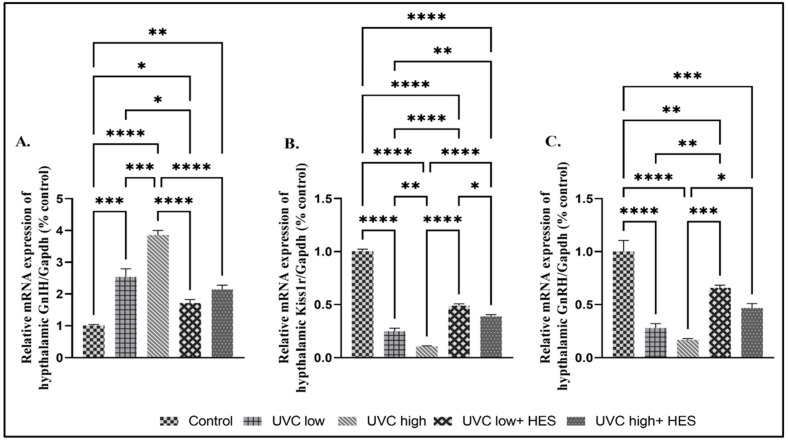
Effects of UVC irradiation, alone or in combination with HES, on the expression levels of reproductive-related genes in the hypothalamus of male rats. Rats exposed to total body irradiation with low or high doses of artificial UVC for 8 h/day for 8 consecutive weeks showed dose-dependent substantial changes in the hypothalamic gene expression of (**A**) gonadotropin-inhibiting hormone (GnIH), (**B**) kisspeptin-1receptor (Kiss1r), and (**C**) gonadotropin-releasing hormone (GnRH). HES significantly reversed the effects caused by exposure to UVC radiation. Data are expressed as mean ± SE from six rats per group. Asterisks indicate a statistically significant difference: **** *p* < 0.0001, *** *p* < 0.001, ** *p* < 0.01, * *p* < 0.05.

The hypothalami of male rats exposed to UVC radiation showed significant upregulation in the mRNA expression level of the GnIH gene, and significant suppression in the mRNA expression levels of the Kiss1 (will be described later), Kiss1r, and GnRH genes, in comparison to values observed in the control group (Figure 6A–C). These results were also noticed in the high-dose-UVC-irradiated group compared to the low-dose-UVC-irradiated group (Figure 6A–C). On the contrary, the hypothalami of irradiated rats pre-treated with HES significantly expressed lower levels of GnIH and higher levels of GnRH, Kiss1r, and Kiss1 than values observed in the UVC-irradiated groups (Figure 6A–C).

### 2.6. Effects of UVC Irradiation, Alone or in Combination with HES, on the Expression Levels of Reproductive-Related Genes in the Pituitary Glands of Male Rats

**Figure 7:**
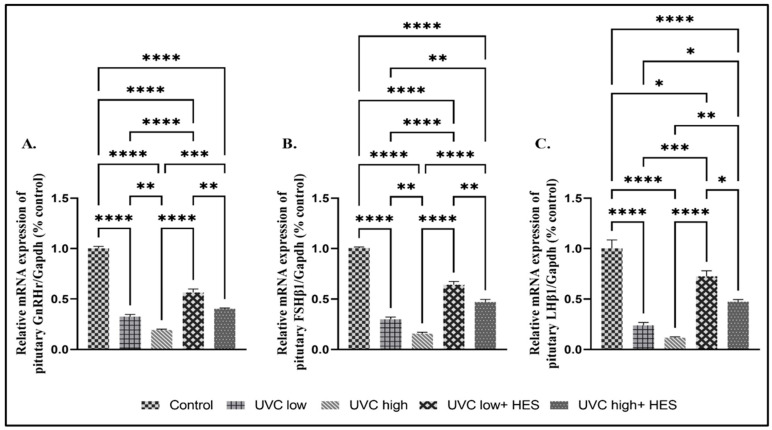
Effects of UVC irradiation, alone or in combination with HES, on the expression levels of reproductive-related genes in the pituitary glands of male rats. Rats exposed to total body irradiation with low or high doses of artificial UVC for 8 h/day for 8 consecutive weeks showed dose-dependent substantial changes in the pituitary gene expression of (**A**) gonadotropin-releasing hormone receptor (GnRHr), (**B**) follicle-stimulate hormone β1 (FSHβ1), and (**C**) luteinizing hormone β1 (LHβ1). HES significantly mitigates the effects caused by exposure to UVC radiation. Data are expressed as mean ± SE from six rats per group. Asterisks indicate a statistically significant difference: **** *p* < 0.0001, *** *p* < 0.001, ** *p* < 0.01, * *p* < 0.05.

Significant downregulation was detected in the mRNA expression levels of the GnRHr, FSHβ1, and LHβ1 genes in the UVC-irradiated rats in comparison to the control group (Figure 7A–C). The high-dose-UVC-irradiated group displayed significant downregulation in the expression levels of the pituitary GnRHr, FSHβ1, and LHβ1 genes compared to those of the low-dose-UVC-irradiated group (Figure 7A–C). In contrast, UVC-irradiated rats pretreated with HES revealed significant upregulation of the mRNA expression levels of the GnRHr, FSHβ1, and LHβ1 genes compared to those of the UVC-irradiated groups (Figure 7A–C).

### 2.7. Effects of UVC Irradiation, Alone or in Combination with HES, on the Expression of Kiss 1 and Steroidogenic Genes in the Testicles of Male Rats

**Figure 8:**
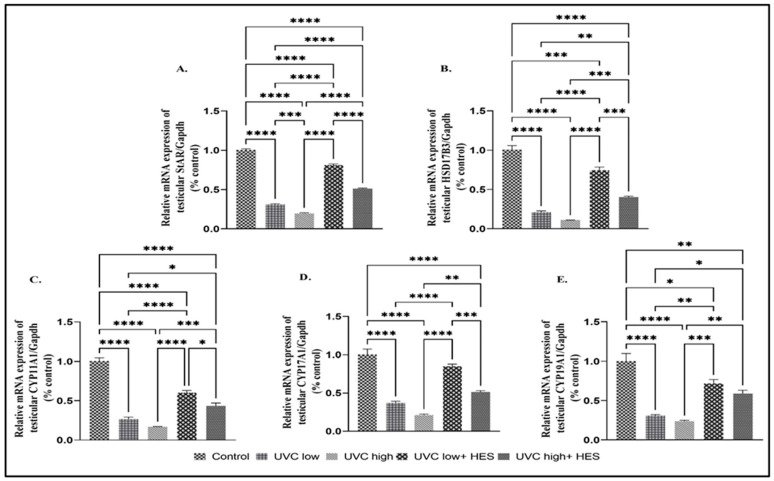
Effects of UVC irradiation, alone or in combination with HES, on the expression of steroidogenic enzymes in testicles of male rats. Expression levels of testicular steroidogenic genes (**A**) steroidogenic acute regulatory (StAR), (**B**) hydroxysteroid dehydrogenase-3-beta 17 (HSD17B3), (**C**) cytochrome P450 family 11 subfamily A member 1 (CYP11A1), (**D**) cytochrome P450 family 17 subfamily A member 1 (CYP17A1), and (**E**) cytochrome P450 family 19 subfamily A member 1 (CYP19A1) were evaluated using real-time PCR analysis in control and UVC-irradiated rats with or without HES pretreatment. Data are expressed as mean ± SE from six rats per group. Asterisks indicate a statistically significant difference: **** *p* < 0.0001, *** *p* < 0.001, ** *p* < 0.01, * *p* < 0.05.

UVC-irradiated rats exhibited a significant downregulation in the mRNA expression of the testicular Kiss 1 ( will be described later) and steroidogenic genes (StAR, HSD17β3, CYP11A1, CYP17A1, and CYP19A1), relative to the control group (Figure 8A–E). Conversely, testicles of UVC-irradiated rats pretreated with HES expressed significant upregulation of the mRNA levels for these steroidogenic genes, compared to those of UVC-irradiated groups (Figure 8A–E).

### 2.8. Effects of UVC Irradiation, Alone or in Combination with HES, on Testicular Autophagy-Related Genes Expression in Male Rats

**Figure 9:**
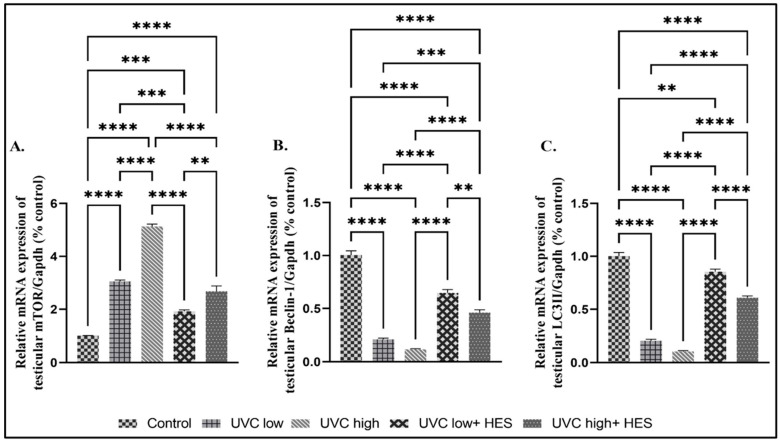
Effects of UVC irradiation, alone or in combination with HES, on testicular autophagy-related gene expression in male rats. Expression of autophagy-related genes [(**A**) mammalian target of rapamycin (mTOR), (**B**) Beclin1, and (**C**) microtubule-associated protein-light chain 3 II (LC3II)] were examined using real-time PCR analysis in testes of control and UVC-irradiated rats, with or without HES pretreatment. Data are expressed as mean ± SE from six rats per group. Asterisks indicate a statistically significant difference: **** *p* < 0.0001, *** *p* < 0.001, ** *p* < 0.01.

**Figure 10:**
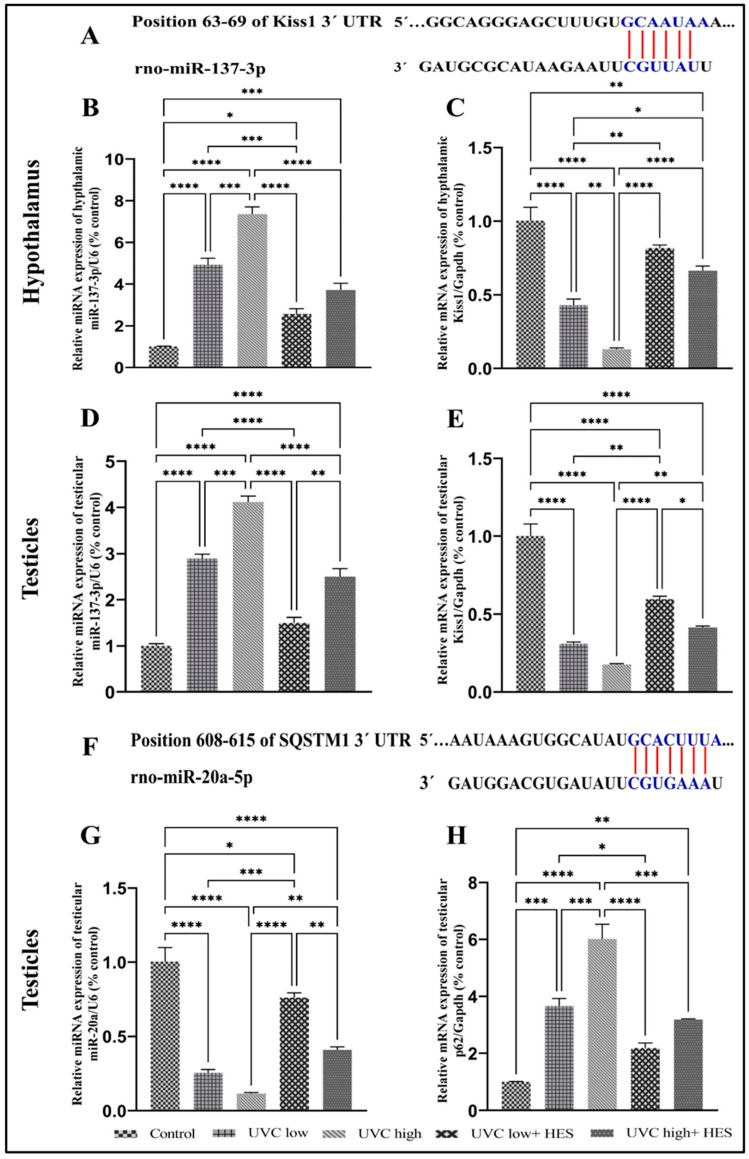
Effects of UVC irradiation alone or in combination with HES on the gene expression of miR- 20a and miR-137-3p and their potential targets in male rats. Bioinformatic predictions of SQSTM1/p62 and Kiss1 regulation by miRNAs. (**A**,**F**) Schematic representation of predicted binding sites for miR-137-3p and miR-20-5p in rat 3’UTR of Kiss1 gene and SQSTM1/p62, respectively. Seed regions of miR-20-5p and miR-137-3p are highlighted in red (A and F). Expression of hypothalamic and testic-ular miR-137-3p (**B**,**D**) and its target Kiss1 (**C**,**E**), as well as testicular miR-20-5p (**G**) and its target SQSTM1/p62 (**H**) were examined using real-time PCR analyses in control and UVC-irradiated rats with or without HES pretreatment. Data is expressed as mean ± SE from six rats per group. Asterisks indicate a statistically significant difference: **** *p* < 0.0001, *** *p* < 0.001, ** *p* < 0.01, * *p* < 0.05.

Significant upregulations in the testicular autophagy-related gene expression levels of mTOR (Figure 9A) and p62 (Figure 10H) and remarkable reductions of the Beclin1 and LC3II expression levels were seen in UVC-irradiated rats, relative to the control group (Figure 9A–C). On the contrary, pretreatment with HES significantly decreased the rat testicular expression of the mTOR gene (Figure 9A) and p62 (Figure 10H) and significantly increased testicular Beclin1 and LC3II expression levels compared to the UVC-irradiated rats (Figure 9A–C).

### 2.9. Effects of UVC Irradiation, Alone or in Combination with HES, on the Gene Expression of miR-20a and miR-137-3p and Their Targets in Male Rats

Two online miRNA target databases, TargetScan and miRTarBase, were utilized to predict potential targets of miR-20a-5p and miR-137-3p. The results identified SQSTM1/p62 as a target gene for miR-20a-5p and Kiss1 as a target gene for miR-137-3p. As illustrated in Figure 10A,F, the sequences from positions 63 to 69 of the Kiss1 3’ UTR were found to be complementary to miR-137-3p, while the nucleotide sequences from positions 608 to 615 of the SQSTM1/p62 3’ UTR were found to be complementary to miR-20a-5p.

UVC radiation led to a notable dose-dependent upregulation of miR-137-3p and a decrease in Kiss1 gene expression in both the hypothalamus (Figure 10B,C) and the testicles (Figure 10D,E) of rats relative to the control group. Among the UVC-irradiated groups, rats that received high-dose treatments showed marked upregulation in the hypothalamic and testicular expression levels of miR-137-3p (Figure 10B,D) and Kiss1 (Figure 10C,E), compared to those of the low-dose-irradiated group. Moreover, UVC radiation caused a significant dose-dependent downregulation of miR-20a and an upregulation of p62 gene expression in the testicles of rats, compared to the control group (Figure 10G,H). High-dose-UVC-irradiated rats exhibited significant upregulation in the testicular expression levels of p62 (Figure 10H), compared to those of the low-dose-irradiated group.

In contrast, UVC-irradiated rats pre-treated with HES displayed significant downregulation of miR-137-3p, while increasing Kiss1 gene expression was observed in both the hypothalamus and testicles of these rats, compared to the UVC-irradiated groups (Figure 10B–E). Furthermore, HES treatment resulted in significant increases in miR-20a levels, while decreasing p62 gene expression in testes, compared to the UVC-irradiated rats (Figure 10G,H).

### 2.10. Correlation Study

We observed several significant correlations among the various parameters studied under the influences of low- and high-dose UVC radiation in male rats. Table 1 represents the effects of exposure to a low dose of UVC radiation.

### 2.11. Agonistic Interaction of HES with Reproductive and Autophagic Receptors in Male Rats

**Figure 11:**
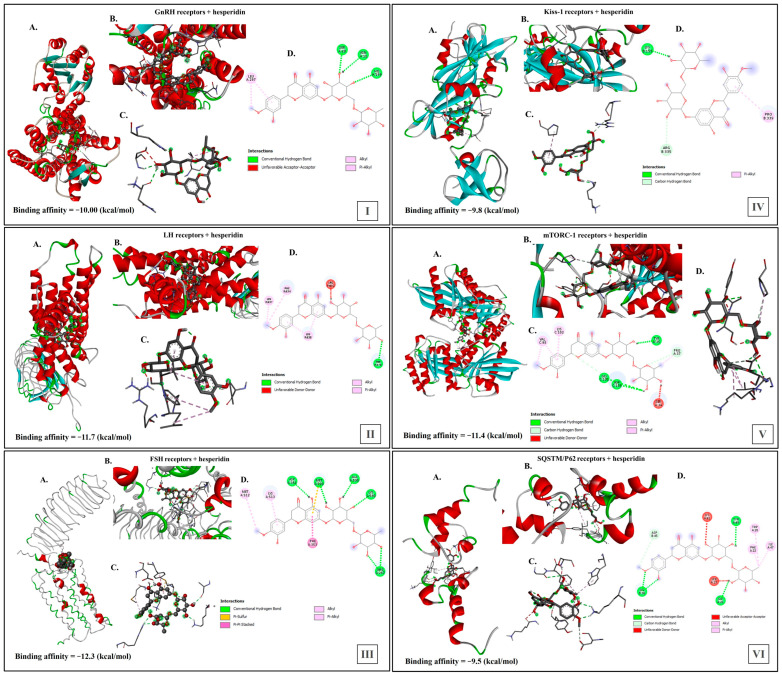
Molecular docking studies of HES against reproductive and autophagic receptors in male rats. Docking studies were conducted on the active sites of six target receptor proteins [(**I**) GnRHr, (**II**) LHr, (**III**) FSHr, (**IV**) Kiss1r, (**V**) mTORC1r, and (**VI**) SQSTM/p62] with HES: (A) 3D images showing the binding affinity of HES with each receptor; (B) 3D images showing the binding pockets between HES and each receptor; (C) 3D images showing the types of interactions between HES and the receptor protein residues; and (D) 2D images showing different types of bonds and interactions between HES and the assessed receptor protein.

Docking studies were conducted on the active sites of six target receptor proteins, GnRHr, LHr, FSHr, Kiss1r, mTORC1r, and SQSTM/p62, with HES. The docking interactions of these targets with HES are detailed in Figure 11. The docking scores and interaction analyses for HES indicate its potential to bind to multiple targets associated with reproduction and autophagy.

As shown in Figure 11(I), HES bound with GnRHr, with a binding affinity score of (−10 kcal/mol), by conventional hydrogen bonds at interaction sites of asparagine (ASN) (A:18), serine (SER) (A:20), and histidine (HIS) (A:199), and by alkyl and Pi-Alkyl bonds at leucine (LEU) (A:297); additionally, the presence of unfavorable acceptor–acceptor interactions was determined.

As shown in Figure 11(II), HES bound with LHr, with a binding affinity score of (−11.7 kcal/mol), by conventional hydrogen bonds at interaction sites of threonine (THR) (R:379), and by Alkyl and Pi-Alkyl bonds at phenylalanine (PHE) (R:634) and leucine (LEU) (R:637 and R:638); additionally, the presence of unfavorable donor–donor interactions at arginine (ARG) (R:632) was determined.

Figure 11(III) revealed that HES bound with FSHr, with a binding affinity score (−12.3 kcal/mol), by conventional hydrogen bonds at interaction sites of aspartic acid (ASP) (A:351), methionine (MET) (A:520), alanine (ALA) (A:595), lysine (LYS) (A:598), and arginine (ARG) (A:247), as well as by Pi-Sulfur bonds at MET (A:520), by Pi-Pi Stacked bonds at phenylalanine (PHE) (A:353), and by alkyl and Pi-Alkyl bonds at LYS (A:513) and MET (A:512).

HES bound with Kiss1r, with a binding affinity score (−9.8 kcal/mol), by conventional hydrogen bonds at interaction sites of lysine (LYS) (B:155), by carbon hydrogen bonds at arginine (ARG) (B:335), and by Pi-Alkyl bonds at proline (PRO) (B:339) (Figure 11(IV)).

Moreover, HES bound with mTORC-1r, with a binding affinity score (−11.4 kcal/mol), by conventional hydrogen bonds at interaction sites of tyrosine (TYR) (A:35), glycine (GLY) (C:101), and aspartic acid (ASP) (C:105), as well as by carbon hydrogen bonds at proline (PRO) (A:37), and by Alkyl and Pi-Alkyl bonds at valine (VAL) (C:98) and lysine (LYS) (C:102); additionally, the presence of unfavorable donor–donor interactions at serine (SER) (A:34) (Figure 11(V)) was determined.

As illustrated in Figure 11(VI), HES bound with SQSTM/p62, with a binding affinity score (−9.5 kcal/mol), by conventional hydrogen bonds at interaction sites of glycine (GLY) (A:21), lysine (LYS) (B:36) and tyrosine (TYR) (A:49), as well as by carbon hydrogen bonds at aspartic acid (ASP) (B:45), and by Alkyl and Pi-Alkyl bonds at phenylalanine (PHE) (A:22), tryptophan (TRP) (A:28), and isoleucine (ILE) (A:47); additionally, the presence of unfavorable acceptor–acceptor interactions and unfavorable donor–donor interactions at serine (SER) (A:23) and lysine (LYS) (A:51) was determined.

## 3. Discussion

A substantial body of research has highlighted the harmful effects of various types of radiation on male fertility; however, nothing is known about the impacts of ultraviolet type-C radiation on the male reproductive physiology and the underlying regulatory mechanisms in animals. This gap underscores the need to investigate how UVC exposure may affect male reproductive health.

### 3.1. Influence of UVC Radiation on Male Reproductive Physiology

The mature male testis has two fundamental roles: the production of sperm and the secretion of sex steroid hormones. The present work explored the effects of UVC radiation on testicular morphology, sperm quality, reproductive hormones of the hypothalamic–pituitary–testicular axis, and testicular steroidogenic genes in male rats. The results demonstrated that exposure to UVC radiation triggered reductions in the testicular weight and gonadosomatic index of male rats. The degree of atrophy increased with an increase in irradiation dose; specifically, a high dose of UVC radiation resulted in a decrease in the net testicular weight and gonadosomatic index in male rats. This suggests a subsequent decline in testosterone levels and a deterioration in sperm quality.

Assessment of sperm parameters is the most crucial clinical laboratory test used to evaluate male fertility. Our results established the existence of a dose-dependent response to UVC irradiation that markedly diminished the sperm quality, as UVC-irradiated male rats exhibited reductions in waving and progressive sperm motility, elevation in the non-progressive sperm motility, diminutions in live sperm percentage and overall sperm count, an increased % of abnormal sperms, and altered morphology.

The intricate hormonal communication implicated in the hypothalamic–pituitary–testicular axis, particularly GnRH, GnIH, FSH, FSHβ1, LH, LHβ1, testosterone, and Kiss1, plays a fundamental role in male reproductive functions. Nonetheless, the effects of UVC radiation exposure on these hormones remain largely unexplored. Among the hormones and receptors assessed herein, our results demonstrated that, compared to controls, male rats irradiated with UVC exhibited significant reductions in serum concentrations of FSH, LH, and free testosterone; significant downregulations in hypothalamic gene expression levels of Kiss1, Kiss1r, and GnRH; marked upregulation in the expression level of hypothalamic GnIH; and significant downregulations in gene expression levels of GnRHr, FSHβ1, and LHβ1 in the pituitary gland and the testicular Kiss1. Simultaneously, our findings implied that UVC radiation exposure directly impacts the synthesis and secretion of reproductive-related hormones in the hypothalamus, pituitary gland, and testes, in a dose-dependent manner, in male rats.

Hormones play a key role in the functionality of the male reproductive system. The testicular functions depend on the hypothalamic–pituitary–gonadal axis. GnRH and GnIH are secreted from the hypothalamus. GnRH is the gatekeeper of mammalian reproductive development and function. The synthesis and release of GnRH are regulated by hypothalamic kisspeptin neurons. GnRH stimulates anterior pituitary gonadotrophs to secrete gonadotropins (LH and FSH), which are essential for regulating testicular activities. FSH stimulates Sertoli cell function and spermatogenesis, while LH acts on Leydig cells to produce testosterone.

FSH and LH synthesis are regulated at the transcriptional level by FSHβ and LHβ gene expression, respectively. The production of the distinct β-subunit is crucial for controlling FSH and LH synthesis [20]. FSHβ and LHβ confer the specific actions of the gonadotropins. Similar to FSH and LH secretion, the transcription of the gonadotropin β subunits is also regulated by GnRH [20].

Kisspeptin and GnIH are members of the RFamide peptide family, and their distinct opposing roles highlight their significance in regulating reproductive activities. These neuropeptides act as key modulators, influencing reproductive functions in response to internal and external cues. The Kiss1/Kiss1r system is a crucial mediator and potent stimulator of the reproductive function of the hypothalamus–pituitary–testicular axis that governs male fertility [21]. Research has highlighted potential regulatory roles for kisspeptin in spermatogenesis, modulation of sperm function, testicular size, steroidogenesis, and testosterone secretion [22,23]. The results of the present study demonstrated that low levels of circulating gonadotropins and testosterone, along with decreased expression of the pituitary (FSHβ1 and LHβ1) and hypothalamic (GnRH and GnRHr) genes, as well as reductions in sperm quantity, were associated with the decreased expression levels of Kiss1 and Kiss1r in the hypothalamus and testes of rats exposed to UVC radiation.

In contrast to kisspeptin, GnIH inhibits GnRH and consequently, suppresses the hypothalamic–pituitary–testicular axis [24]. Reproductive-related peptide-3 (RFRP-3), the mammalian ortholog of GnIH, directly or indirectly inhibits GnRH synthesis and secretion from the hypothalamus in mammals [25]. GnIH also suppresses kisspeptin secretion, which is responsible for stimulating GnRH release from the hypothalamus [24,25]. GnIH can inhibit the HPG axis by inhibiting GnRH and/or kisspeptin neuron expression [24,25]. Mice subcutaneously injected with GnIH exhibited a significant decrease in LH and testosterone concentrations, along with marked reductions in the hypothalamic expression of GnRH and Kiss1, as well as decreased pituitary expression levels of FSHβ and LHβ and declines in testicular expression of the StAR and 3β-HSD proteins [26]. Furthermore, RFRP3 was found to induce a reduction in germ cell survival and proliferation and negatively affect testicular testosterone synthesis by inhibiting the dose-dependent expression of steroidogenic factors such as StAR, CYP11A1, and 3β-HSD in mice [27]. This is consistent with our findings, which revealed that rats exposed to UVC radiation showed significant downregulation of key steroidogenic genes in the testes (StAR, CYP11A1, CYP17A1, CYP19A1, and HSD17β3), leading to impaired testicular steroidogenesis and a dose-dependent reduction in testosterone secretion.

Collectively, the data from the present study support the role of GnIH in the modulation of male reproductive functions by the inhibition of the hypothalamic–pituitary–testicular (HPT) axis through the suppression of GnRH and kisspeptin expression. We observed that exposure to UVC radiation resulted in upregulation of GnIH gene expression in adult male rats, which was associated with the inhibition of the Kiss1/Kiss1r signaling pathway and downstream HPT activity. These findings provide valuable insights into stress-related reproductive dysfunction and infertility. Thus, our data suggest that GnIH may act as a key mediator of stress effects on mammalian reproduction, possibly contributing to infertility.

### 3.2. UVC Radiation Induces Oxidative Stress

Exposure to UVC radiation can impair physiological cellular functions by inducing an increased formation of reactive oxygen species (ROS), leading to oxidative stress [28]. Oxidative stress occurs when there is an imbalance between ROS generation and the body’s antioxidant defense mechanisms [28]. Our findings indicated that UVC exposure significantly reduced antioxidant enzyme levels while increasing lipid peroxidation in male rats, highlighting the role of free radicals in the mediation of damage following UVC exposure. The data showed a dose-dependent relationship between UVC exposure and testicular oxidative damage biomarkers, evidenced by reductions in SOD and TAC, alongside increases in MDA. This suggests that UVC-induced oxidative stress generates free radicals that cause significant harm to testicular tissue. These changes can adversely affect sperm motility, concentration, morphology, and viability, ultimately contributing to male infertility.

In the male reproductive tract, oxidative stress and the generation of ROS have been linked to impaired sperm parameters, including motility, morphology, viability, and count, and positively correlated with infertility [29]. It has been shown that TAC and SOD were positively associated with sperm motility, viability, concentration, and morphology [30,31]. Our results coincided with those of [12], who showed that UVC irradiation of human sperm resulted in decreased sperm motility and viability. This reduction is likely linked to UVC-induced oxidative stress.

ROS generated by exposure to UVC radiation not only adversely affects sperm quality but also disorients the reproductive hormonal profiles. Our findings demonstrated that exposure to UVC irradiation induces stress, resulting in increased expression of the hypothalamic GnIH gene. This increase downregulates Kiss1, Kiss1r, GnRH, GnRHr, gonadotropin synthesis and secretion, and circulating testosterone levels. Consequently, these effects impaired spermatogenesis and deteriorated sperm quality, ultimately suppressing the male reproductive functions in rats.

Oxidative stress negatively affects male reproductive functions and may induce infertility, either directly or indirectly, by affecting the HPG axis and/or disrupting its crosstalk with other hormonal axes [32]. Increasing evidence suggests that stress reduces kisspeptin levels while elevating GnIH expression and activity, disrupting the HPT axis and leading to reproductive disorders in male rats [33]. Stressful conditions have also been shown to increase RFRP-3 expression, further contributing to HPT dysfunction and suppression of reproductive activity in adult male rats [25].

### 3.3. UVC Radiation Induces Disruption of Autophagy

Autophagy is an evolutionarily conserved lysosome-mediated catabolic process essential for maintaining cellular homeostasis and cell survival [14]. The autophagic process involves distinct steps, which include induction, recognition, selection of cargo, autophagosome formation, autophagosome–lysosome fusion, lysosomal degradation, and nutrient recycling [14].

The role of autophagy in UV-induced male infertility is largely unexplored. Thus, we investigated UVC radiation’s effect on autophagy in male rats. The mTOR, Beclin1, LC3II, and p62 genes are central autophagy-related genes involved in the autophagy flux. The mTOR and p62 genes have been described as major negative regulators of autophagy [34,35]. P62 is associated with the degradation of autophagosomes [36]. However, Beclin1 and LC3II are indispensable autophagy-promoting genes that positively regulate the formation of autophagosomes [37,38]. Beclin1 regulates the phosphatidylinositol-3-kinase complex, which is important to initiate the autophagic process [14].

In the present study, male rats exposed to total body irradiation with low and high doses of UVC for 8 h/day for 8 consecutive weeks showed dose-dependent effects on the mRNA expression levels of mTOR, Beclin1, LC3II, and p62 in the rats’ testicles. Our results revealed significant upregulations of mTOR and p62 gene expression in the testes of UVC-irradiated rats, alongside notable reductions in Beclin1 and LC3II expression, compared to the control group. These findings consistently indicate that autophagy activity is inhibited in the testes of male rats following chronic UVC exposure. Autophagy influences multiple aspects of the male reproductive system. Under physiological conditions, it plays a vital role in ensuring testicular cell integrity and maintaining spermatogenesis, sperm quality, and steroidogenesis in testicular tissue [14]. Recently, the relationship between chronic stress and autophagy has been focused on. Increasingly, evidence has emerged to verify the theory that chronic stress suppresses autophagy [4,39,40]. The present study revealed negative correlations between oxidative stress markers and mTOR and p62, while showing positive correlations with Beclin1 and LC3II. Additionally, there were positive correlations between the lipid peroxidation marker and mTOR and p62, whereas negative correlations were observed with Beclin1 and LC3II. Consequently, it was determined that UVC-induced ROS suppressed the autophagy pathway in male rats. Therefore, we verified that exposure of male rats to UVC radiation disrupts the autophagy mechanism, leading to significant reductions in testosterone secretion, impaired spermatogenesis, and decreased sperm quality. This could prove a clear link between impaired autophagy activity and male infertility in this context. Additionally, the results revealed that GnIH mRNA expression exhibited a negative correlation with Beclin1 and LC3II while showing a positive relation with mTOR and p62 gene expression levels. These data suggested that GnIH exerts suppressive effects on male reproduction in UVC-irradiated rats, likely due to its role in mediating the inhibition of autophagy in the testes.

### 3.4. UVC Radiation Alters miRNA Expression Patterns

The present study proved the existence of a dose-dependent response as to the effect of UVC radiation on miRNA expression patterns in rats. Our real-time PCR revealed that exposure to UVC radiation induced a significant downregulation of testicular miR-20a-5p expression levels and a remarkable upregulation in the expression levels of miR-137-3p in the hypothalamus and testes of rats relative to the control. Interestingly, using an in silico search, we found that miR-20a-5p targets the position of 608–615 of SQSTM1/p62 3’UTR, while miR-137-3p targets the position of 63–69 of Kiss1 3’UTR. This result implies the possible roles of these miRNAs in UVC radiation-induced male infertility and autophagy suppression.

Recently, evidence indicated that miRNAs exhibited different expression levels under oxidative stress [41]. Our study evidenced that the distinct expression patterns of miR-20a-5p and miR-137-3p induced by UVC radiation significantly correlated with the assessed oxidative stress markers. This indicated that the miR-20a-5p and miR-137-3p expression levels were dysregulated by UVC-induced oxidative stress and highlighted their possible roles as key mediators in UVC-induced oxidative stress in male rats.

MiR-20a is a member of the miR-17-92 family. In adult mouse testes, disruption of the miR-17-92 cluster resulted in severe testicular atrophy, empty seminiferous tubules, decreased sperm production, and impaired spermatogenesis in mice [42]. MiR-20a was also positively correlated with FSH and LH in men [43]. These data were consistent with our results which showed significant correlations between miR-20a levels and the reproductive parameters in male rats exposed to UVC radiation, indicating a potential role for miR-20a in male fertility.

MiR-137 suppressed the gonadotropins and testosterone in male rats by targeting Kiss1 [44,45]. In the present study, the silencing of Kiss1 gene expression by miR-137-3p, as detected by the in silico analysis, and the significant elevation in miR-137 expression alongside the notable reduction in Kiss1 gene expression in the hypothalamus and testicles of UVC-irradiated male rats compared to controls, as detected by real-time PCR analysis, could prove the crucial role of miR-137 in mediating reproductive dysfunction induced by UVC exposure through the downregulation of the Kiss1 gene in male rats. Thus, dysregulation of miR-20a-5p and miR-137-3p may negatively affect the fertility of male rats exposed to UVC radiation by interfering with reproductive hormone synthesis and secretion, testicular function, and sperm quality. Consequently, these microRNAs may serve as important biomarkers for diagnosing male infertility and assessing spermatogenesis.

Remarkably, miRNAs act as new players in regulating reproduction and autophagy. The absence of the testicular miR-17-92 cluster impaired spermatogenesis in mice by activating the mTOR signaling pathway [42]. Moreover, Ref. [46] revealed that overexpression of miR-137 led to a decrease in LC3 expression and an increase in SQSTM1/p62. It is currently unknown whether miR-20a-5p and miR-137-3p can influence the autophagy regulatory network in male rats exposed to UVC irradiation. The present study highlights the importance of the interplay between autophagy and the microRNAs in the regulation of male reproductive health. We have shown that in silico search detected p62 as a potential target for miR-20a-5p. Also, the decreased level of miR-20a-5p in the testicles of UVC-irradiated rats negatively correlated with mTOR and p62, while positively correlating with LC3 and Beclin1. Therefore, this decrease in miR-20a-5p levels failed to suppress p62 gene expression in the testicles of UVC-irradiated rats, leading to its upregulation. Conversely, the increased miR-137-3p levels in the hypothalamus and testicles positively correlated with mTOR and p62, while negatively correlating with LC3 and Beclin1 in the UVC-irradiated rat testicles. These data proved that dysregulation of miR-20a-5p and miR-137-3p in the UVC-irradiated rats may subsequently impair the autophagy process and result in male fertility in UVC-irradiated rats.

### 3.5. Hesperidin Alleviates UVC-Induced Reproductive Damage in Male Rats

One of the strategies used to mitigate radiation-induced damage is using radioprotectors to safeguard against such damage. Research on the relationship between HES and male reproduction is limited. Notably, only one study has investigated this connection in the context of radiation, demonstrating that HES administered before γ-radiation effectively protected rat testicles against apoptosis and oxidative stress [47]. However, the protective effects of HES against UVC irradiation have not been previously investigated. The present study investigates the in silico interactions of HES with several receptors, including FSHr, LHr, Kiss-1r, mTORC-1r, and SQSTM/p62, in male rats. This metric is critical for assessing the affinity and potential efficacy of HES in modulating receptor activity. A lower docking score indicates a more favorable binding mode in a protein–ligand complex, suggesting stronger interactions between the ligand and the receptor. Docking analyses demonstrated strong binding affinities between HES and these targets, suggesting its potential as a protective agent for male reproductive health against UCV radiation. These findings indicate that HES may be beneficial in mitigating the adverse effects of radiation exposure, thereby promoting reproductive health and reducing the detrimental impacts of UVC exposure. The results of the present study indicate HES’s potential to activate pathways such as GnRHr, FSHr, LHr, and Kiss1r, while inhibiting mTORC-1r and SQSTM/p62. Ultimately, these results highlight the possible role of HES in safeguarding male reproductive health in the face of UVC exposure.

Our findings demonstrated that pretreatment with HES provided a protective effect by reversing the adverse outcomes and restoring the affected parameters to near-normal levels in UVC-irradiated male rats. In the present study, HES effectively mitigated UVC-induced damage by scavenging ROS. This was achieved through the enhancement of SOD and TAC levels, alongside a reduction in MDA levels. In agreement with our results, HES has been shown to alleviate UVA and UVB radiation-induced skin damage by counteracting oxidative stress in human keratinocytes [48,49].

Regarding the effect of HES on autophagy, we showed that HES activated autophagic markers in the testes of UVC-irradiated rats by inhibiting mTOR and p62 while inducing Beclin1 and LC3II. This is consistent with the findings of [50,51] who demonstrated that HES can trigger and enhance the autophagy process in response to stress and diseases.

It has been reported that natural products can regulate several miRNAs, targeting many downstream signaling genes to control different cell functions through the natural-product–miRNA–downstream axis. To the best of our knowledge, this study is the first to demonstrate the effects of HES pretreatment on the expression levels of miRNAs (miR-20a and miR-137) in UVC-irradiated rats. We showed that pretreatment with HES significantly alleviated the effects of UVC exposure on the expression levels of miR-20a and miR-137 in rats by increasing miR-20a expression and decreasing miR-137 expression, which in turn promoted autophagy, and improved the male fertility issues.

Natural products have dual functions, improving radio-sensitization in cancer cells and radioprotection in non-cancer cells. The radioprotective mechanism of these natural products through miRNA modulation has not yet been described in detail. It has been shown that miR-20a-5p promoted radio-resistance in nasopharyngeal cancer cells [52]. Moreover, miR-137 enhanced the sensitivity of cancer cells to irradiation [53]. Our study supports the finding that pretreatment with HES significantly modulates the expression of miR-20a and miR-137 in UVC-irradiated rats. Particularly, HES pretreatment led to a boost in miR-20a levels and a decrease in miR-137 levels, compared to the UVC-irradiated group. This alteration in radiation-associated miRNAs may contribute to hesperidin’s radioprotective effects, potentially enhancing the radio-resistance of cells. Therefore, targeting miR 20a and miR-137 with HES is an ideal strategy to combat UVC radiation.

Based on the findings in this study, we revealed that HES improves UCV radiation-induced male reproductive defects. Our results indicated that the potential therapeutic strategy of HES involves mitigating the adverse effects of UVC irradiation on male reproductive physiology, possibly through its antioxidant activities, autophagy-induction properties, functional improvement of testicles, improved sperm parameters, enhancements of testicular steroidogenesis, restoration of the reproductive hormonal changes in the HPT axis, and the targeting of miR 20a and miR-137. Therefore, HES may be utilized as a suitable radioprotector agent for workers and the public in dealing with UVC radiation.

## 4. Conclusions

The present study elucidates the detrimental effects of UVC radiation on male reproductive physiology in rats and thoroughly investigates the underlying mechanisms and potential protective strategies. Understanding these effects is crucial for assessing risks, particularly in environments with prevalent UVC exposure, such as certain medical and industrial settings. Our findings underscore the critical roles of oxidative stress, autophagy, reproductive hormones, and microRNAs in UVC-induced reproductive challenges. Rats exposed to UVC radiation exhibited significant dysregulation of testicular steroidogenesis, impaired spermatogenesis, deteriorated sperm quality, and altered reproductive hormonal profiles, leading to a decline in fertility. Our data indicate that the suppression of autophagy, stimulation of GnIH, and alteration of microRNAs serve as key mediators of UVC-induced stress effects on mammalian reproduction, potentially contributing to male infertility. Furthermore, our research explores the potential protective effects of HES in mitigating the adverse impacts of UVC irradiation on male reproductive physiology. Given these findings, it is imperative to implement strict safety measures when working with UVC sources, including the use of protective gear and avoiding direct exposure. These precautions are essential to safeguard reproductive health in environments where UVC exposure is a concern.

## 5. Materials and Methods

### 5.1. Animals

A total of 30 healthy male adult Sprague Dawley rats (weighing 200–220 g) were used in this study. Rats were acclimatized for two weeks and maintained on a basal diet with free access to running water under controlled environmental conditions at room temperature, 25 ± 2 °C. Rats were randomly divided into one control and four experimental groups, as described above. Rats were obtained from the laboratory animal unit of the School of Veterinary Medicine, Badr University, Cairo, Egypt. The experimental protocol used in the present study was approved by the Institutional Animal Care and Use Committee of Zagazig University (approval number: ZU-IACUC/2/F/136/2024).

### 5.2. UVC Source

The radiation sources used were UVC lamps (model 3222-638-78092, PHILIPS, made in Poland) of 8 watts in power, measuring 30 cm in length, and emitting radiation at a wavelength of 254 nm. The lamps were positioned 30 cm above the rat’s cage. The experimental setup involved a timer and a power supply controller. The timer controlled the UV exposure time, setting it as 8 h/day. During exposure, a power supply controller monitored and regulated the electrical supply’s stability. The average doses of UVC radiation exposure throughout the 8 h/day for the duration of the experimental period of eight weeks were, based on measurements, determined to be 641.41 for the low dose and 1924.23 J/cm^2^ for the high dose, according to the following formula: UV dose (J/cm^2^) = UV intensity (W/ cm^2^) × Exposure time (seconds), as previously described in [54,55].

### 5.3. Experimental Design

Rats were randomly equally distributed into 5 groups of 6 rats each. Group I did not receive irradiation or hesperidin and served as a control group. Groups II and III were exposed to low doses (one UV lamp), whereas Groups IV and V were exposed to high doses (three UV lamps) of artificial UVC radiation for 8 h per day for 8 consecutive weeks. Additionally, before daily UVC exposure, rats in Groups III and V received a 50 mg/kg single daily intraperitoneal injection of hesperidin (Sigma-Aldrich Company, St. Louis, MO, USA), [56]. Hesperidin powder CAS NO 520-26-3, at minimum 80% purity, was purchased from and manufactured by Sigma-Egypt. It was kept below 30 °C in a dry, dark place until it was needed for the experiment.

### 5.4. Sampling

At the end of the experiment, 24 h after the last HES dose, blood samples were obtained from the rat tail vein for the control and irradiated groups. For serum preparation, the samples were centrifuged at 3000 r.p.m for 10 min., then stored at −20 °C for hormone analysis. All rats were subjected to euthanization by decapitation under anesthesia with an intraperitoneal injection of 100 mg/kg thiopental sodium. The testes, pituitary glands, and hypothalamus were rapidly removed and washed with physiological saline. Specimens of the right testis, pituitary glands, and hypothalamus were immediately frozen with liquid nitrogen and preserved at −80 °C for gene expression analysis, whereas further testis specimens were homogenized in ice-cold phosphate buffer saline (0.1M, pH = 7.4) with a glass homogenizer, followed by centrifugation at 10,000× *g* for 30 min at 4 °C. The supernatant was then collected and kept at −20 °C to determine the oxidative stress and lipid peroxidation parameters. For the histopathological and immunohistochemical evaluations, the left testis of each rat in the different experimental groups were fixed in Bouin’s solution for 24 h at room temperature.

### 5.5. Measurement of Testicular Weight and Gonadosomatic Index of Rats

At the end of the experiment, the net testicular weight (g) and gonadosomatic index of each rat were recorded. The testes were rapidly removed, washed with physiological saline, and weighed. The gonadosomatic index % was calculated using the following formula: (gonadosomatic index: GSI = [testis weight/body weight] × 100).

### 5.6. Assessment of Sperm Parameters

Semen samples were evaluated as described previously by [57]. Briefly, the cauda epididymis of one testis/rat was macerated in a sterilized Petri dish containing 2 mL preheated (37 °C) physiological saline.

#### 5.6.1. Sperm Motility

To assess the sperm motility, a drop of previously freshly prepared semen sample was dropped on a clean glass slide, covered by a glass cover slide, and examined under both the low power objective (×10) of a light microscope (to evaluate the waving mass motility of the spermatozoa), and the high power (×40) objective (to estimate the individual progressive and non-progressive motility). The average motility score was recorded in various microscopic fields.

#### 5.6.2. Sperm Vitality

To determine sperm vitality, a smear of one drop of freshly prepared semen sample was mixed with the eosin–nigrosine stain on a glass slide. Spermatozoa with dark pink heads were considered dead, whereas spermatozoa with unstained heads were considered to be alive. Live sperm cells were counted under the light microscope and the number expressed as a percentage.

#### 5.6.3. Sperm Count

Sperm cells were counted using an improved Neubauer hemocytometer after the dilution of semen samples with physiological saline at a rate of 1:4 and the addition of 5 drops of formalin (40%) solution. Sperm concentration/mL = (dilution factor × sperm count in 5 secondary squares × 0.05 × 106), which is usually expressed in terms of sperm × 106/mL.

#### 5.6.4. Sperm Morphology

Sperm morphology was assessed using eosin–nigrosine-stained slides observed under an oil immersion lens. Morphological abnormalities were calculated as a percentage of the total number of counted sperm.

### 5.7. Measurement of Oxidative Stress Biomarkers in Rat Testis Homogenates

Levels of superoxide dismutase (SOD), and total antioxidant capacity (TAC), as well as malondialdehyde (MDA; a marker of lipid peroxidation) levels, were determined in the rat testicular tissues using the commercially available colorimetric assay kits (Catalog No.: SD2521; TA2513; and MD2529, respectively; Bio-diagnostic Company, Giza, Egypt) according to the manufacturer’s instructions.

### 5.8. Measurement of Reproductive Hormones in Male Rats

Measurement of testosterone, follicle-stimulating hormone (FSH), and luteinizing hormone (LH) concentrations were assessed in the rat serum by rat enzyme-linked immunosorbent assay (ELISA) kits (Catalog No. MBS704301; MyBioSource, San Diego, CA, USA), (Catalog No. E-EL-R0391; Elabscience, Houston, TX, USA), (Catalog No. MBS764675, MyBioSource, San Diego, CA, USA), respectively, following the manufacturer’s instructions.

### 5.9. Real-Time Polymerase Chain Reaction

Quantitative real-time polymerase chain reactions (qRT-PCR) were performed for the quantification of the mRNA expression levels of the hypothalamic genes [gonadotropin-inhibiting hormone (GnIH), gonadotropin-releasing hormone (GnRH), Kisspeptin (Kiss1), and Kisspeptin-1receptor (Kiss1r)] and pituitary genes [gonadotropin-releasing hormone receptor (GnRHr), follicle-stimulate hormone β1 (FSHβ1), and luteinizing hormone β1 (LHβ1)], as well as the testicular Kiss1 and steroidogenic genes [steroidogenic acute regulatory (StAR), cytochrome P450 family 11 subfamily A member 1 (CYP11A1), cytochrome P450 family 17 subfamily A member 1 (CYP17A1), cytochrome P450 family 19 subfamily A member 1 (CYP19A1), and hydroxysteroid dehydrogenase-3-beta 17 (HSD17B3)] and the autophagy-related genes in testes [mammalian target of rapamycin (mTOR), Beclin1, microtubule-associated protein-light chain 3 II (LC3II), and p62].

Total RNA was extracted from the hypothalamus, pituitary, and testis using Trizol reagent (Invitrogen; Thermo Fisher Scientific, Inc., Waltham, MA, USA) following the manufacturer’s instructions. Reverse transcription of total RNA into cDNA was performed using the High-Capacity cDNA Reverse Transcription Kit (Applied Biosystems™, Waltham, MA, USA) following the manufacturer’s guidelines. The primer sequences for the target genes and housekeeping genes were synthesized by Sangon Biotech (Beijing, China), and are listed in Table 2.

Reverse transcription of miR-20a-5p, miR-137-3p, and U6 was performed using the stem–loop primer sequences provided in Table 1, following the manufacturer’s instructions, with the miScript II Reverse Transcription Kit (Qiagen, Santa Clarita, CA, USA). The microRNA primers were designed using the online tool available at http://www.srnaprimerdb.com (accessed on 1 March 2024), and based on the mature microRNA sequences obtained from the miRNA database at https://www.mirbase.org/ (accessed on 1 March 2024).

Real-time quantitative PCR was conducted using a Rotor-Gene Q 2plex Real-Time PCR System (Qiagen, Germany) with TOPreal™ qPCR 2X PreMIX (SYBR Green with low ROX) (Enzynomics, Daejeon, South Korea), following the manufacturer’s protocols. The PCR cycling conditions consisted of an initial denaturation at 95 °C for 12 min, followed by 40 cycles of denaturation at 95 °C for 20 s, annealing at 60 °C for 30 s, and extension at 72 °C for 30 s. A melting-curve analysis was performed after amplification. The relative changes in gene expression from the real-time quantitative PCR experiments were calculated using the 2^−ΔΔCT^ method. The results are presented as fold changes in the expression of the examined genes, normalized to the internal housekeeping genes (GAPDH for mRNA and U6 for miRNA), and relative to the normal control group.

### 5.10. Bioinformatics Analysis

To identify the putative miRNA target, we utilized the online miRNA target analysis tools TargetScan (http://www.targetscan.org/ (accessed on 1 March 2024)) and miRTarBase (https://awi.cuhk.edu.cn/~miRTarBase/miRTarBase_2025/php/index.php (accessed on 1 March 2024)) to perform in silico search for targets of miR-20a-5p and miR-137-3p.

### 5.11. Molecular Docking Assessment

The RCSB Protein Data Bank (RCSB PDB; https://www.rcsb.org/ (accessed on 1 March 2024)) and AlphaFold (https://alphafold.ebi.ac.uk/ (accessed on 1 March 2024)) were used to retrieve protein and ligand data, while Molecular Operating Environment (MOE 2022.02, Chemical Computing Group, Montreal, QC, Canada) software was employed for molecular docking. The three-dimensional structure of hesperidin was obtained from the PubChem database in SDF format and imported into MOE for energy minimization and docking with target proteins.

Three-dimensional structures of various rat proteins, including GnRHr, Kiss1r, follicle-stimulating hormone receptor (FSHr), luteinizing hormone receptor (LHr), mammalian target of rapamycin receptor (mTORC-1r), and sequestosome/p62 receptor (SQSTM/p62), were retrieved from the RCSB Protein Data Bank and AlphaFold databases. These target proteins were prepared for docking in MOE (MOE 2015.10) by removing water and ligand molecules and minimizing their energy. The target proteins were docked with the ligands by identifying the binding sites and employing the induced fit model for docking. The resulting protein–ligand interactions were visualized using the same software.

### 5.12. Statistical Analysis

The data were assessed for normality before analysis. One-way analysis of variance (ANOVA) was conducted for statistical analysis, followed by Tukey’s post hoc test for multiple comparisons between experimental groups, using GraphPad Prism 9.5.1 (San Diego, CA, USA). The relationship between variables was analyzed using Pearson’s bivariate correlation test. The *p* values <0.05 were considered statistically significant. Different symbols above the bars indicate statistical significance. Results are presented as the mean ± standard error (SE).

## Figures and Tables

**Table 1:** Correlations between the various parameters examined under the effect of low-dose UVC radiation in male rats. Abbreviations: T., Testosterone; Hypoth., Hypothalamus; Tes. w., Testicular weight; Sp., Sperm; Ab. Sp., Abnormal sperm; Prog. Sp. Motility, Progressive sperm motility.

Parameter		SOD	TAC	MDA	FSH	FSHβ1	LH	LHβ1	T.	GnIH	GnRH	Kiss1	Kiss1r	mTOR	P62	Beclin1	LC3II	miR-20a-5p	miR-137-3p Hypoth.	miR- 137-3p Testes	Tes. W.	Sp. count	Live. Sp.	Ab. Sp.	Prog. Sp. Motility
**SOD**	r *p*	1	0.974 0.001	−0.890 0.017	0.914 0.010	0.911 0.011	0.88 0.01	0.89 0.01	0.933 0.006	−0.867 0.025	0.895 0.016	0.978 0.001	0.842 0.035	−0.818 0.046	−0.991 0.000	0.902 0.014	0.917 0.010	0.880 0.028	−0.971 0.001	−0.839 0.037	0.872 0.023	0.873 0.022	0.855 0.030	−0.857 0.029	0.907 0.012
**TAC**	r *p*	0.974 0.001	1	−0.947 0.004	0.967 0.001	0.952 0.003	0.972 0.001	0.960 0.002	0.975 0.001	−0.975 0.001	0.974 0.001	0.988 0.000	0.962 0.002	−0.947 0.004	−0.953 0.003	0.990 0.000	0.940 0.005	0.954 0.003	−0.993 0.000	−0.962 0.002	0.972 0.001	0.968 0.001	0.968 0.001	−0.966 0.001	0.987 0.000
**MDA**	r *p*	−0.890 0.017	−0.947 0.004	1	−0.951 0.003	−0.940 0.005	−0.929 0.00	−0.927 0.007	−0.902 0.014	0.950 0.004	−0.943 0.004	−0.885 0.019	−0.960 0.002	0.991 0.000	0.909 0.012	−0.941 0.005	−0.974 0.001	−0.927 0.008	0.871 0.024	0.977 0.001	−0.973 0.001	−0.972 0.001	−0.983 0.000	0.985 0.000	−0.963 0.002
**FSH**	r *p*	0.914 0.010	0.967 0.001	−0.951 0.003	1	0.997 0.001	0.940 0.005	0.953 0.003	0.968 0.001	−0.949 0.004	0.955 0.002	0.973 0.001	0.942 0.004	−0.964 0.002	−0.975 0.001	0.952 0.003	0.939 0.005	0.940 0.005	−0.947 0.004	−0.949 0.004	0.969 0.001	0.973 0.001	0.969 0.001	−0.975 0.001	0.988 0.000
**FSHβ1**	r *p*	0.911 0.011	0.952 0.003	−0.940 0.005	0.997 0.001	1	0.922 0.008	0.950 0.003	0.955 0.003	−0.925 0.008	0.946 0.004	0.967 0.001	0.926 0.008	−0.949 0.004	−0.966 0.002	0.933 0.006	0.924 0.008	0.935 0.006	−0.933 0.006	−0.933 0.007	0.958 0.002	0.965 0.002	0.955 0.003	−0.964 0.001	0.975 0.001
**LH**	r *p*	0.882 0.019	0.972 0.001	−0.929 0.007	0.940 0.005	0.922 0.008	1	0.965 0.002	0.962 0.002	−0.964 0.002	0.972 0.001	0.947 0.004	0.969 0.001	−0.935 0.006	−0.958 0.003	0.986 0.000	0.981 0.000	0.970 0.001	−0.963 0.002	−0.970 0.001	0.977 0.001	0.970 0.001	0.975 0.001	−0.974 0.001	0.972 0.001
**LHβ1**	r *p*	0.891 0.015	0.960 0.002	−0.927 0.007	0.953 0.003	0.950 0.003	0.965 0.002	1	0.919 0.009	−0.915 0.010	0.994 0.000	0.956 0.002	0.972 0.001	−0.927 0.008	−0.960 0.001	0.970 0.001	0.953 0.003	0.998 0.000	−0.952 0.003	−0.969 0.001	0.984 0.000	0.987 0.000	0.971 0.001	−0.971 0.001	0.959 0.002
**T.**	r *p*	0.933 0.006	0.975 0.000	−0.902 0.013	0.968 0.001	0.955 0.003	0.962 0.002	0.919 0.009	1	−0.965 0.001	0.9267 0.008	0.972 0.001	0.913 0.011	−0.926 0.008	−0.965 0.002	0.953 0.003	0.932 0.007	0.911 0.011	−0.967 0.002	−0.921 0.009	0.940 0.005	0.936 0.006	0.943 0.004	−0.949 0.003	0.977 0.001
**GnIH**	r *p*	−0.867 0.025	−0.975 0.001	0.950 0.003	−0.949 0.003	−0.925 0.009	−0.964 0.002	−0.915 0.010	−0.965 0.002	1	−0.943 0.004	−0.938 0.005	−0.962 0.002	0.975 0.001	0.954 0.003	−0.979 0.001	−0.959 0.003	−0.914 0.011	0.949 0.004	0.968 0.002	−0.964 0.002	−0.954 0.003	−0.974 0.001	0.970 0.001	−0.984 0.000
**GnRH**	r *p*	0.895 0.015	0.974 0.001	−0.943 0.004	0.955 0.002	0.946 0.004	0.972 0.001	0.994 0.000	0.926 0.008	−0.943 0.004	1	0.959 0.002	0.989 0.000	−0.949 0.004	−0.976 0.001	0.987 0.000	0.962 0.002	0.994 0.000	−0.962 0.002	−0.986 0.000	0.993 0.000	0.993 0.000	0.984 0.000	−0.981 0.000	0.973 0.001
**Kiss1**	r *p*	0.978 0.001	0.988 0.001	−0.885 0.019	0.973 0.001	0.967 0.002	0.947 0.004	0.956 0.002	0.972 0.001	−0.938 0.005	0.959 0.002	1	0.928 0.007	−0.916 0.010	−0.996 0.000	0.963 0.002	0.905 0.013	0.944 0.004	−0.991 0.000	−0.928 0.007	0.951 0.003	0.952 0.003	0.942 0.005	−0.944 0.004	0.973 0.001
**Kiss1r**	r *p*	0.842 0.035	0.962 0.002	−0.968 0.001	0.942 0.004	0.926 0.008	0.969 0.001	0.972 0.001	0.913 0.011	−0.962 0.002	0.989 0.000	0.928 0.007	1	−0.973 0.001	−0.954 0.003	0.987 0.000	0.977 0.001	0.975 0.001	−0.937 0.005	−0.999 0.000	0.994 0.000	0.990 0.000	0.993 0.000	−0.987 0.000	0.972 0.001
**mTOR**	r *p*	−0.818 0.046	−0.947 0.004	0.991 0.002	−0.964 0.002	−0.949 0.004	−0.935 0.006	−0.927 0.007	−0.926 0.008	0.975 0.001	−0.949 0.003	−0.916 0.010	−0.973 0.001	1	0.938 0.005	−0.959 0.002	−0.963 0.002	−0.922 0.009	0.906 0.012	0.980 0.006	−0.975 0.001	−0.973 0.001	−0.986 0.000	0.985 0.000	−0.980 0.001
**P62**	r *p*	−0.99 0.001	−0.953 0.003	0.909 0.012	−0.975 0.001	−0.966 0.002	−0.958 0.002	−0.968 0.001	−0.965 0.002	0.954 0.003	−0.976 0.001	−0.996 0.000	−0.954 0.003	0.938 0.005	1	−0.979 0.001	−0.816 0.048	−0.959 0.003	0.992 0.000	0.953 0.003	−0.875 0.022	−0.875 0.022	−0.859 0.028	0.861 0.027	−0.912 0.011
**Beclin1**	r *p*	0.902 0.014	0.990 0.001	−0.941 0.005	0.952 0.003	0.933 0.006	0.986 0.001	0.970 0.001	0.954 0.003	−0.979 0.001	0.987 0.000	0.963 0.002	0.987 0.000	−0.959 0.002	−0.979 0.000	1	0.968 0.002	0.971 0.001	−0.976 0.001	−0.986 0.000	0.988 0.000	0.981 0.000	0.985 0.000	−0.980 0.001	0.984 0.000
**LC3II**	r *p*	0.917 0.010	0.940 0.005	−0.974 0.001	0.939 0.005	0.924 0.008	0.981 0.001	0.953 0.003	0.932 0.006	−0.959 0.002	0.962 0.002	0.905 0.013	0.977 0.001	−0.963 0.002	−0.816 0.047	0.968 0.002	1	0.960 0.002	−0.911 0.012	−0.982 0.000	0.986 0.000	0.981 0.000	0.985 0.000	−0.979 0.001	0.981 0.000
**miR-** **20a-5p**	r *p*	0.880 0.020	0.954 0.003	−0.927 0.007	0.940 0.005	0.935 0.006	0.970 0.001	0.998 0.000	0.911 0.011	−0.914 0.011	0.994 0.000	0.944 0.005	0.975 0.001	−0.922 0.009	−0.959 0.002	0.971 0.001	0.960 0.002	1	−0.946 0.004	−0.971 0.001	0.984 0.000	0.986 0.000	0.971 0.001	−0.970 0.001	0.954 0.003
**miR-137-3p** **Hypoth.**	r *p*	−0.971 0.001	−0.993 0.001	0.871 0.023	−0.947 0.004	−0.933 0.006	−0.963 0.002	−0.952 0.003	−0.967 0.001	0.949 0.004	−0.962 0.002	−0.991 0.000	−0.937 0.005	0.906 0.013	0.992 0.000	−0.976 0.001	−0.910 0.011	−0.946 0.004	1	0.934 0.006	−0.950 0.004	−0.946 0.004	−0.940 0.005	0.938 0.005	−0.966 0.001
**miR-137-3p** **Testes**	r *p*	−0.839 0.037	−0.962 0.002	0.977 0.001	−0.949 0.003	−0.933 0.006	−0.970 0.001	−0.969 0.001	−0.921 0.008	0.968 0.001	−0.986 0.000	−0.928 0.007	−0.999 0.000	0.980 0.001	0.953 0.003	−0.986 0.000	−0.982 0.000	−0.971 0.001	0.934 0.006	1	−0.995 0.000	−0.991 0.000	−0.996 0.000	0.992 0.000	−0.978 0.001
**Tes. w.**	r *p*	0.872 0.023	0.972 0.001	−0.973 0.001	0.969 0.001	0.958 0.002	0.977 0.001	0.984 0.000	0.940 0.005	−0.964 0.002	0.993 0.000	0.951 0.003	0.994 0.000	−0.975 0.001	−0.875 0.022	0.988 0.000	0.986 0.000	0.984 0.000	−0.950 0.004	−0.995 0.000	1	0.999 0.000	0.997 0.000	−0.996 0.000	0.996 0.000
**Sp.** **count**	r *p*	0.873 0.022	0.968 0.001	−0.972 0.001	0.973 0.001	0.965 0.002	0.970 0.001	0.987 0.000	0.936 0.006	−0.954 0.003	0.993 0.000	0.952 0.003	0.990 0.000	−0.973 0.001	−0.875 0.022	0.981 0.000	0.981 0.000	0.986 0.000	−0.946 0.004	−0.991 0.000	0.999 0.000	1	0.995 0.000	−0.995 0.000	0.995 0.000
**Live Sp.**	r *p*	0.855 0.030	0.968 0.001	−0.983 0.000	0.969 0.001	0.955 0.003	0.975 0.001	0.971 0.001	0.943 0.004	−0.974 0.001	0.984 0.000	0.942 0.005	0.993 0.000	−0.986 0.000	−0.859 0.028	0.985 0.000	0.985 0.000	0.971 0.001	−0.940 0.005	−0.996 0.000	0.997 0.000	0.995 0.000	1	−0.999 0.000	0.999 0.000
**Ab. Sp.**	r *p*	−0.857 0.029	−0.966 0.001	0.985 0.000	−0.975 0.001	−0.964 0.001	−0.974 0.001	−0.971 0.001	−0.949 0.003	0.970 0.001	−0.981 0.000	−0.944 0.004	−0.987 0.000	0.985 0.000	0.861 0.027	−0.980 0.001	−0.979 0.001	−0.970 0.001	0.938 0.005	0.992 0.000	−0.996 0.000	−0.995 0.000	−0.999 0.000	1	−0.989 0.000
**Prog. Sp. Motility**	r *p*	0.907 0.012	0.987 0.000	−0.963 0.002	0.988 0.000	0.975 0.001	0.972 0.001	0.959 0.002	0.977 0.001	−0.984 0.000	0.973 0.001	0.973 0.001	0.972 0.001	−0.980 0.001	−0.912 0.011	0.984 0.000	0.981 0.000	0.954 0.003	−0.966 0.001	−0.978 0.001	0.996 0.000	0.995 0.000	0.999 0.000	−0.989 0.000	1

**Table 2:** Primers used for quantitative real-time PCR.

Gene Name	Forward Primer (5′-3′)	Reverse Primer (5′-3′)	Product Size	Accession No.
**Primers for mRNAs**				
**GnIH**	AGAGCAACCTAGGAAACGGGTGTT	AGGACTGGCTGGAGGTTTCCTATT	84	NM_023952.1
**GnRH**	AGGAGCTCTGGAACGTCTGAT	AGCGTCAATGTCACACTCGG	100	NM_012767.2
**GnRHr**	TCAGGACCCACGCAAACTAC	CTGGCTCTGACACCCTGTTT	182	NM_031038.3
**Kiss1**	TGCTGCTTCTCCTCTGTGTGG	ATTAACGAGTTCCTGGGGTCC	110	NM_181692.1
**Kiss1r**	CTTTCCTTCTGTGCTGCGTA	CCTGCTGGATGTAGTTGACG	102	NM_023992.1
**LHβ1**	AGAATGGAGAGGCTCCAGGG	CCATGCTAGGACAGTAGCCG	187	NM_012858.2
**FSHβ1**	ACCAGCTTTCTCTCCCATGC	GAGAAGCAGGGGGTCCTAGA	171	NM_001007597.2
**STAR**	CCCAAATGTCAAGGAAATCA	AGGCATCTCCCCAAAGTG	187	NM_031558.3
**HSD17β3**	AGTGTGTGAGGTTCTCCCGGTACCT	TACAACATTGAGTCCATGTCTGGCCAG	161	NM_054007.1
**CYP11A1**	AAGTATCCGTGATGTGGG	TCATACAGTGTCGCCTTTTCT	127	NM_017286.3
**CYP17A1**	TGGCTTTCCTGGTGCACAATC	TGAAAGTTGGTGTTCGGCTGAAG	90	NM_012753.2
**CYP19A1**	GCTGAGAGACGTGGAGACCTG	CTCTGTCACCAACAACAGTGTGG	178	NM_017085.2
**mTOR**	GCAATGGGCACGAGTTTGTT	AGTGTGTTCACCAGGCCAAA	94	NM_019906.2
**P62**	GGAAGCTGAAACATGGGCAC	CCAAGGGTCCACCTGAACAA	183	NM_181550.2
**Beclin1**	GAATGGAGGGGTCTAAGGCG	CTTCCTCCTGGCTCTCTCT	180	NM_001034117.1
**LC3-II**	GAAATGGTCACCCCACGAGT	ACACAGTTTTCCCATGCCCA	147	NM_012823.2
**GAPDH**	GGCACAGTCAAGGCTGAGAATG	ATGGTGGTGAAGACGCCAGTA	143	NM_017008.4
**Primers for miRNAs and internal control**				
**miR-137-3p**	AGCCAGCGTTATTGCTTAAGAAT	GTCGTATCCAGTGCAGGGT		
**miR-20a-5p**	AAGCGCCTTAAAGTGCTTATAGT	GTCGTATCCAGTGCAGGGT		
**U6**	GCTCGCTTCGGCAGCACA	GAGGTATTCGCACCAGAGGA		
**Stem-loop primers for miRNAs and internal control**				
**miR-137-3p**	5′-GTCGTATCCAGTGCAGGGTCCGAGGTATTCGCACTGGATACGACCTACGC-3′			
**miR-20a-5p**	5′-GTCGTATCCAGTGCAGGGTCCGAGGTATTCGCACTGGATACGACCTACCT-3′			
**U6**	5′-AACGCTTCACGAATTTGCGTG-3′			

## Data Availability

The original contributions presented in this study are included in the article. Further inquiries can be directed to the corresponding authors.
